# An integrated, mobile service for diabetic retinopathy in rural India

**Published:** 2011-09

**Authors:** Mohita Sharma, Anindya S Chakrabarty, Rathi Pavan, RC Sharma, Goel Pratibha

**Affiliations:** Nayantara project lead and chief ophthalmologist, Tiupati Eye Centre, C-8, Sector 19, Noida 201301, India; Senior eye consultant, Tiupati Eye Centre, C-8, Sector 19, Noida 201301, India; Project Manager, Tiupati Eye Centre, C-8, Sector 19, Noida 201301, India; Director, Tiupati Eye Centre, C-8, Sector 19, Noida 201301, India; Senior eye consultant, Tiupati Eye Centre, C-8, Sector 19, Noida 201301, India

**Figure:**
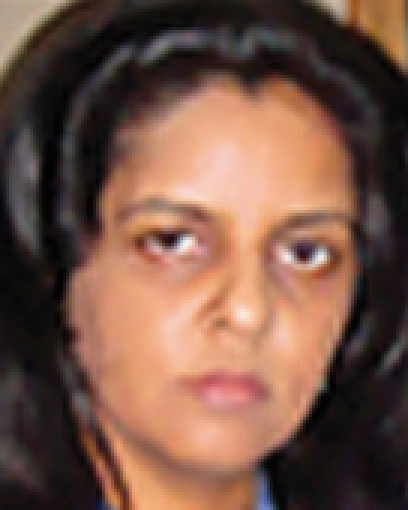


**Figure:**
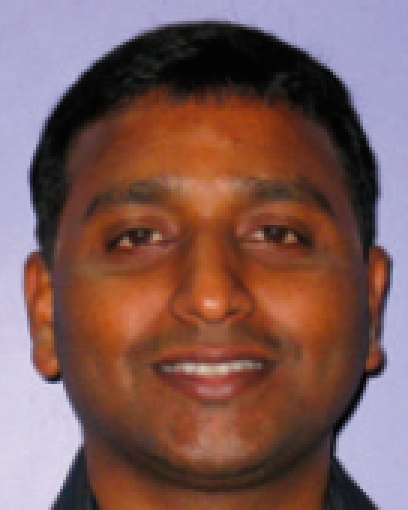


**Figure:**
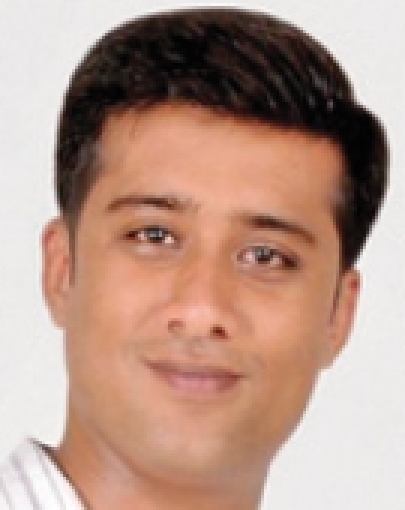


**Figure:**
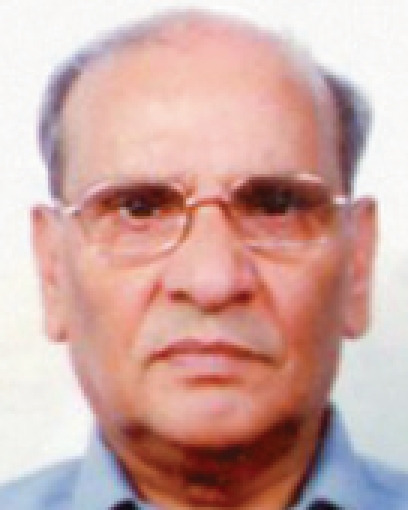


**Figure:**
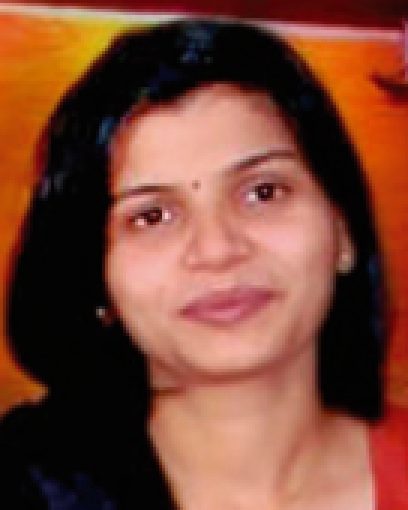


The prevalence of diabetes among adults aged 30 years and above in rural India is 13.2%,^1^ nearly double the global average for 2010. How can these individuals best be detected, treated and followed up? Rural communities have limited access to medical services, which leads to poor control of diabetes and hypertension. As a result, diabetic complications such as diabetic retinopathy (DR) may also be more frequent in rural than in urban areas.

In order to effectively address DR in rural India, we must:

- Find people with diabetes
- Examine their retinas
- Provide laser treatment if needed, often enough to halt the progression of DR and preserve sight
- Follow up those treated as well as those not treated on a regular basis
- Give advice on how people can prevent the complications of diabetes, including retinopathy.

This can be very difficult to achieve in rural India, where patients are likely to live far from treatment centres. Travel may be difficult and expensive, and can also lead to loss of earnings. This means that, even if treatment were free, patients may not come for yearly retinal examinations and those who need laser may not attend for follow-up or repeated treatment as often as needed.

**Figure:**
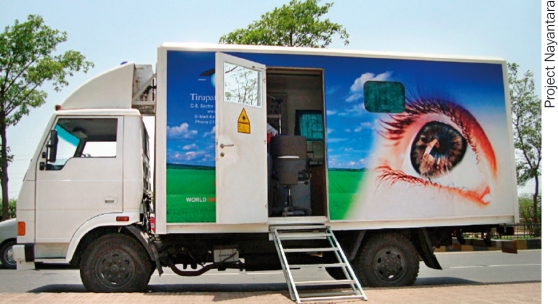
The specially designed mobile unit is air conditioned and fully equipped with a slit lamp, visual field analyser, digital fundus camera, laser, and generator. INDIA

In rural areas, local physicians will care for people with diabetes, but many of them will not have the skills or the equipment to detect and refer patients with suspected DR. Many ophthalmologists may also not be confident in examining retinas and interpreting the findings, and the majority will not have skills in photocoagulation nor access to a laser. However, these individuals are an invaluable resource in the community: after training and capacity building, they have the potential to play a vital and cost-effective role in a DR programme.

Project Nayantara (in Hindi, ‘Nayan' means eye and ‘tara’ means star) was devised as an integrated approach to address obstacles faced by patients as well as the limitations of local health professionals.

The project has a mobile examination and treatment unit (Figure 1), which contains a fundus camera and a laser. This unit, staffed by an ophthalmologist and allied personnel, travels to pre-determined locations in five districts in Uttar Pradesh, visiting each location once a month. This brings a high-quality diagnosis and treatment point very close to patients and does so regularly enough to ensure that patients are able to come for follow-up and repeated laser treatment when needed.

The equipment is only part of the solution, however. Project Nayantara relies on the relationships the team has developed with local physicians, diabetologists, ophthalmologists, and other health care workers. These health professionals refer patients with suspected DR to the mobile unit for examination and treatment: a very efficient use of the mobile team's time and skills.

The team builds local capacity by training the participating ophthalmologists in the use of the equipment and in the diagnosis and treatment of diabetic retinopathy. Training is also given to a variety of health workers, including local health promoters and general physicians. Physicians are taught how to perform a basic eye examination, including identifying signs of diabetic retinopathy using a direct ophthalmoscope. They are also encouraged to buy their own pocket ophthalmoscopes.

**Figure:**
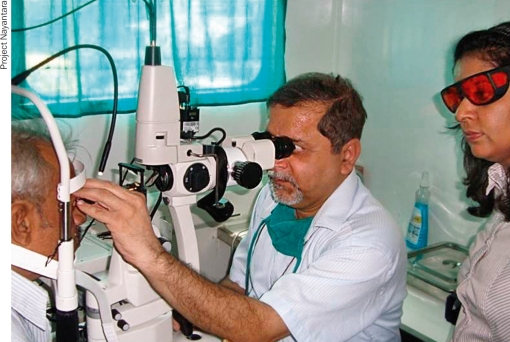
A local ophthalmologist receives hands-on training inside the mobile truck. INDIA

## Financial sustainability

Running costs (approximately US $1,200 per month) are paid for by external funding for the first three years. After this, it is anticipated that costs will be covered by income generated from the mobile unit, making the project financially sustainable.

Treatment is provided free to those who cannot pay and at low cost to those who can. Patients who have a monthly income of less than US $30 are entitled to free treatment (this was true for approximately 60% of those treated in the initial phase of the project). Additional revenue is generated from private patients who are referred for investigations using some of the more sophisticated equipment in the mobile unit, such as scanners that measure body fat.

Any income generated is shared between the local ophthalmologist and the project team: the local ophthalmologist receives 40% as an incentive to ‘earn while they learn’ and the remaining income (60%) contributes towards running costs.

The capital cost of the equipment in the van is approximately US $140,000. This would have to be replaced roughly every seven years and will be paid for by the income generated from the unit in the years thereafter.

## Results

Since Project Nayantara started in July 2010, the van has regularly visited 25 destinations involving 98 ophthalmologists, 142 general practitioners/ physicians and 102 health workers. The team has examined 6,498 diabetes patients who were all referred by local health professionals. The team has completed 2,267 laser procedures and performed fundus fluorescein angiography on 1,827 patients. Each of these examinations and procedures also served as opportunities to train local ophthalmologists.

Of those requiring treatment, 95% have completed three sessions of pan-retinal laser photocoagulation (PRP) on the planned date and 100% have completed three sessions of PRP within three months of the planned date, signifying excellent compliance.

In the first nine months, 126 procedures (including intravitreal injections and vitreoretinal operations) were performed at the base hospital in Delhi as a result of referrals by the project team. These operations have been performed either free of charge or at minimal cost to the patient.

The intention is that, once ophthalmologists and diabetologists are confident and competent in detecting and treating DR in one location, the van will stop regular visits to that particular location and will move on to a new location.

This project, by training physicians and local ophthalmologists, achieves three goals:

- Patients do not have to travel more than 50 km to receive treatment for diabetic retinopathy
- Patients are followed up by a doctor in the same area who is a trusted and familiar caregiver
- After the mobile unit moves on to a new location, any new patients with diabetes will be well managed.

Initial experience with this integrated approach suggests that it increases patient compliance with treatment and follow-up. In addition, it has empowered local communities and health care professionals by transferring skills and building local capacity to diagnose and treat diabetic retinopathy.
